# Finished Genome Sequence of Collimonas arenae Cal35

**DOI:** 10.1128/genomeA.01408-14

**Published:** 2015-01-08

**Authors:** Je-Jia Wu, Victor C. L. de Jager, Wen-Ling Deng, Johan H. J. Leveau

**Affiliations:** a Department of Plant Pathology, University of California, Davis, California, USA; b Agricultural Biotechnology Center, National Chung Hsing University, Taichung, Taiwan; c Ph.D. Program in Microbial Genomics, National Chung Hsing University and Academia Sinica, Taipei, Taiwan; d Department of Microbial Ecology, Netherlands Institute of Ecology (NIOO-KNAW), Wageningen, The Netherlands; e Department of Plant Pathology, National Chung Hsing University, Taichung, Taiwan

## Abstract

We announce the finished genome sequence of soil forest isolate Collimonas arenae Cal35, which comprises a 5.6-Mbp chromosome and 41-kb plasmid. The Cal35 genome is the second one published for the bacterial genus Collimonas and represents the first opportunity for high-resolution comparison of genome content and synteny among collimonads.

## GENOME ANNOUNCEMENT

Collimonads are oligotrophic, chitinolytic, rhizosphere-competent soil bacteria with antifungal, mycophagous, and mineral-weathering properties (1). To date, three species of Collimonas have been described (2, 3), but the only Collimonas genome available is that of the Dutch dune soil isolate C. fungivorans Ter331 (*Cf*Ter331, GenBank accession no. CP002745 for the chromosome and EU315244 for plasmid pTer331). The *Cf*Ter331 genome has been instrumental in describing the Collimonas chitinolytic system (4), the genes underlying the production of antifungal polyyne-like compounds called collimomycins (5), the molecular interactions with model fungus Aspergillus niger (6), and the phenotypic variation among other collimonads isolated from the same dune soil as Ter331 (7). However, with only one Collimonas genome sequence available to date, intrageneric comparison at single-nucleotide resolution has not yet been feasible. Here, we report the finished genome sequence of Collimonas arenae Cal35 (*Ca*Cal35), which was recovered from a forest soil in California (8).

Genomic DNA of *Ca*Cal35 was extracted from an overnight culture using a DNeasy Blood and Tissue kit (QIAGEN, Valencia, CA). A 10-kb PacBio RS II-compatible library was constructed by the UC Davis Genome Center and sequenced on 3 single-molecule real-time (SMRT) cells using P4-C2 chemistry. *De novo* assembly was performed with the help of SMRT Analysis software v2.2.0 (Pacific Biosciences) featuring HGAP 2 (9), and subsequent correction with quiver in addition to Gepard v1.30 (10) to reveal two circular replicons: a 5,603,532-bp chromosome (G+C content 56.15%; 85.54× coverage) and a 41,440-bp plasmid (G+C content 50.54%; 73.21× coverage) which we designated pCal35. Gene prediction by RAST (11) revealed 5,019 coding sequences, 54 tRNA genes, and 3 rRNA operons (5S, 16S, 23S) on the chromosome, in addition to 57 coding sequences on pCal35.

The chromosome of *Ca*Cal35 appears mostly syntenic with that of *Cf*Ter331, with the exception of one 2.75-Mbp inversion centered on the origin of replication. *Ca*Cal35 shares 69% of its genes with Ter331, of which 80% are >80% identical at the predicted amino acid level. The chitinolytic system of *Ca*Cal35 is very similar to that of *Cf*Ter331, but *Ca*Cal35 possesses at least two additional chitinase genes. Clearly missing from the *Ca*Cal35 genome is gene cluster K which is responsible for the production of collimomycins (5–7). Interestingly, in *Cf*Ter331, this gene cluster is located near one of the chromosome inversion points. Unrelated to pTer331 (12), plasmid pCal35 carries several genes with high coding similarity to plasmids from plant-pathogenic species of Xanthomonas (for example, pXAC64, pXCV38, pXcB) and Ralstonia (pRSC35). In addition, it harbors a stretch of DNA with high similarity to plasmid 2 from Polaromonas JS666 and coding for tRNA 2-selenouridine synthase, alkane 1-monooxygenase, and a LuxR-type regulator.

### Nucleotide sequence accession numbers.

The Cal35 chromosome and plasmid sequences are available under GenBank accession numbers CP009962 and CP009963, respectively.
